# The effects of 8 weeks of repeated sprint training on aerobic endurance, anaerobic power, agility and explosive strength in college badminton players

**DOI:** 10.7717/peerj.21111

**Published:** 2026-04-24

**Authors:** Chao Wei, Jing An, Yong Lin, Sen Yang, Qingying Zhu, Lin Zhou

**Affiliations:** 1 School of Physical Education, Shandong University, Jinan, China; 2 Beijing Jiaotong University Weihai, Weihai, China

**Keywords:** Repeated sprint training, High-intensity interval training, Aerobic capacity, Anaerobic power, Agility, Badminton players, Physical education

## Abstract

**Background:**

Badminton is a physically demanding sport requiring a combination of aerobic endurance, anaerobic power, agility, and explosive strength. Repeated sprint training (RST) has emerged as a time-efficient strategy for improving physical performance, but its comprehensive effects on badminton players are not fully elucidated.

**Methods:**

Twenty-eight male collegiate badminton players were randomly assigned to either a repeated sprint training group (RST; *n* = 14) or a high-intensity interval training group (HIIT; *n* = 14). In addition to regular skill-based badminton practice, the RST group performed 30-m all-out running sprints twice per week (2–3 sets of 6 × 30 m, 30 s passive recovery between sprints and 2 min active recovery between sets) over 8 weeks. The HIIT group completed standard badminton training combined with running-based high-intensity intervals prescribed around 90% HR_max. Before and after the intervention, all participants were assessed for aerobic capacity ( ${\dot {\rm V}}$O_2_max, v ${\dot {\rm V}}$O_2_max, first and second ventilatory thresholds), anaerobic power (Wingate peak power (PP), mean power (MP), fatigue index), repeated sprint ability (6 × 30-m sprints: ideal time (IS), total time (TS), performance decrement), agility (modified T-test), and lower-limb power (countermovement and spike jumps).

**Results:** All participants completed the study, and no significant baseline differences were found between groups (*p* > 0.121). Significant main effects of time and group × time interactions were observed for ${\dot {\rm V}}$O_2_max, v ${\dot {\rm V}}$O_2_max, VT_1_, PP, MP, modified agility T-test, and spike jump height. The RST group showed greater post-intervention improvements in these variables than the HIIT group (all *p* ≤ 0.002), whereas the HIIT group demonstrated significant but smaller gains in ${\dot {\rm V}}$O_2_max, v ${\dot {\rm V}}$O_2_max, VT_1_, PP, and spike jump height (*p* < 0.05). Significant time effects were also found for IS, TS, VT_2_, and countermovement jump height, with both groups improving after training (all *p* < 0.05), particularly in the RST group.

**Conclusion:** An 8-week, twice-weekly 30-m RST program added to standard badminton training proved effective for concurrently improving aerobic capacity, anaerobic power, repeated sprint ability, agility, and lower limb explosive power in collegiate male badminton players. These findings suggest that this specific RST protocol is a potent and time-efficient training modality for enhancing the multifaceted physical fitness required for badminton performance.

## Introduction

Badminton is a high-intensity intermittent racket sport characterized by explosive movements, including lunges, jumps, smashes, and rapid changes of direction, interspersed with brief recovery periods (Liu, Abdullah & Abu Saad, 2024). As detailed in a comprehensive review by Phomsoupha & Laffaye (2015), the temporal structure of the game requires players to repeatedly perform maximal efforts with incomplete recovery, placing heavy demands on both the phosphocreatine and glycolytic energy pathways. Success in the sport is contingent not only on technical and tactical proficiency but also on a highly developed physical capacity (Ma et al., 2025). Specifically, elite players have been shown to possess significantly higher anaerobic power and aerobic capacity compared to sub-elite counterparts (Ooi et al., 2009), suggesting that conditioning programs must target a robust aerobic system to sustain performance alongside a powerful anaerobic system to fuel match-winning actions (Bishop & Girard, 2013). Consequently, identifying training methods that simultaneously enhance these diverse physiological attributes is integral to player development.

Traditionally, athlete conditioning has involved high volumes of both sport-specific and general endurance training. However, given the congested competition schedules and technical training demands in modern sports, coaches often seek more time-efficient conditioning strategies. High-intensity interval training (HIIT) and repeated sprint training (RST) have emerged not as replacements for traditional methods, but as potent, time-efficient strategies to induce significant physiological adaptations (Hall et al., 2023). While HIIT is widely recognized for improving cardiorespiratory fitness through submaximal to maximal intervals, RST represents a more extreme intensity domain. RST is characterized by brief, repeated bouts of “all-out” supramaximal exercise interspersed with incomplete recovery periods (Burgomaster et al., 2008). Evidence suggests that RST can elicit adaptations in anaerobic capacity and neuromuscular function that may be superior to those derived from work-matched lower-intensity intervals (Whyte, Gill & Cathcart, 2010).

A growing body of evidence demonstrates the multifaceted effects of RST. Research indicates that RST significantly enhances anaerobic performance, with meta-analyses identifying substantial effect sizes for power-related outcomes (Hall et al., 2023). These improvements are likely underpinned by muscular adaptations, such as increased glycolytic enzyme activity and improved ion regulation (MacDougall et al., 1998). Moreover, despite its anaerobic nature, RST provides a potent stimulus for aerobic capacity, with documented increases in maximal oxygen uptake ( ${\dot {\rm V}}$O_2max_) and citrate synthase activity comparable to traditional endurance training (Burgomaster et al., 2005; Burgomaster, Heigenhauser & Gibala, 2006). These benefits have been observed across various intermittent sports, including soccer (Yan & Li, 2025; Dupont, Akakpo & Berthoin, 2004), basketball (Wang, Wang & Qian, 2025), wrestling (Farzad et al., 2011), and karate (Xu & Wang, 2025).

Despite the theoretical alignment between RST mechanics and badminton physiological demands (Phomsoupha & Laffaye, 2015), its specific application in this sport remains insufficiently examined compared to general HIIT. While systematic reviews confirm that HIIT is effective for improving aerobic indices in racket sports (Liu, Abdullah & Abu Saad, 2024), a specific knowledge gap exists regarding the comparative efficacy of RST *vs* HIIT on the comprehensive anaerobic profile required for badminton. Existing literature has primarily focused on aerobic adaptations (Liu et al., 2021), or agility drills (citations regarding badminton agility/sprint), often lacking a head-to-head comparison between RST and matched HIIT protocols. It remains unclear whether RST can provide “added value” over standard HIIT by concurrently enhancing repeated-sprint ability (RSA), agility, and lower-limb explosive power without compromising aerobic development.

Therefore, the present study aimed to examine the effects of an 8-week RST program compared to a volume-matched HIIT protocol on multiple dimensions of physical performance in collegiate male badminton players. The HIIT protocol was selected as the active comparator because it represents the current evidence-based standard for time-efficient aerobic conditioning in court sports. We hypothesized that while both interventions would improve aerobic capacity, the RST intervention would result in significantly greater improvements in anaerobic power, RSA, agility, and lower-limb explosive strength due to the higher neuromuscular demand of supramaximal sprinting. Furthermore, it was expected that RST would concurrently enhance aerobic capacity, thereby contributing to a more comprehensive improvement in overall physical conditioning status.

## Method

### Participants

**Figure 1:**
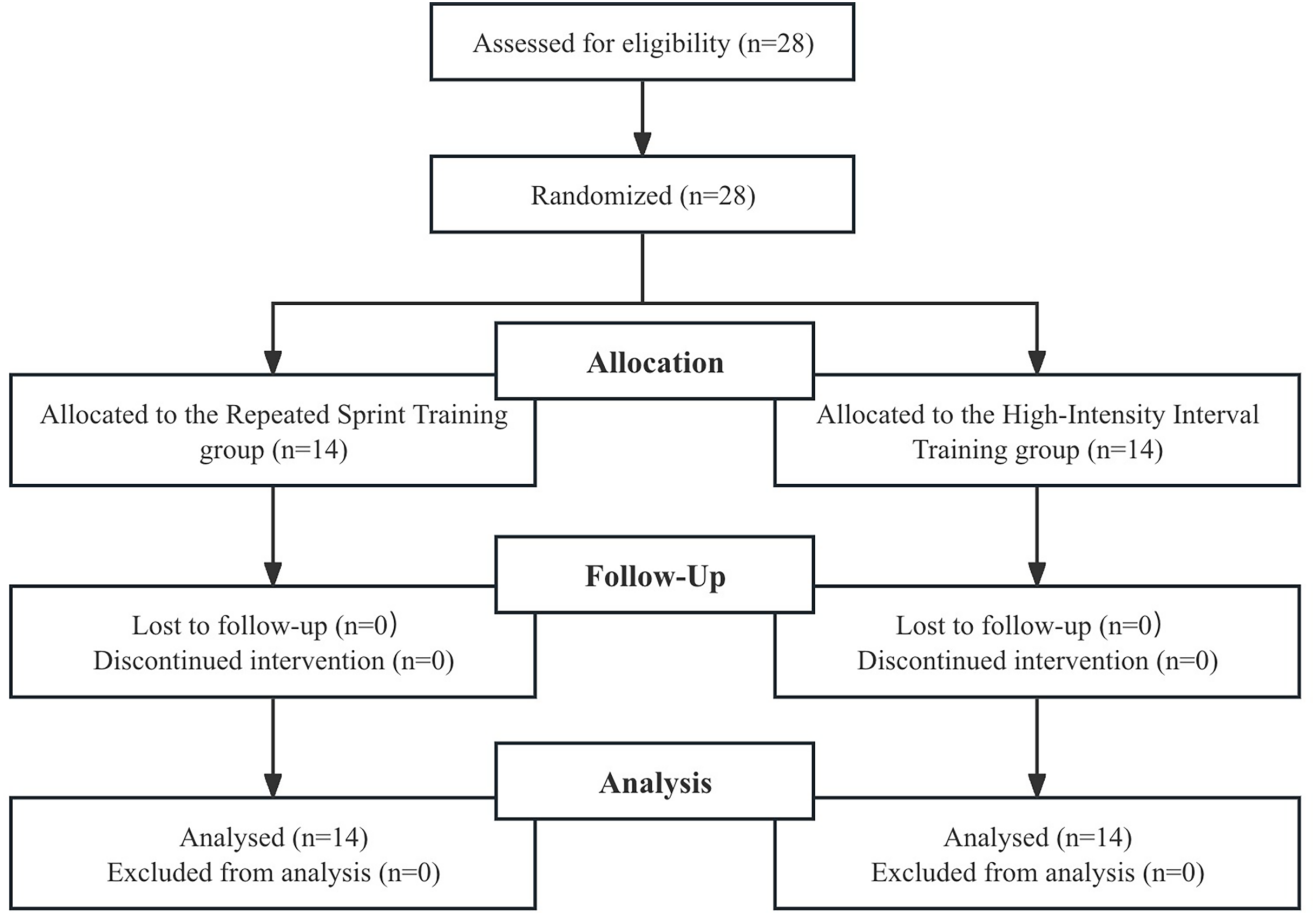
Flow chart of the progress through the phases of the study according to the CONSORT statements.

A total of 28 male collegiate badminton players were recruited to participate in this investigation. To be eligible for inclusion, subjects were required to be active members of university teams competing at the provincial level and possess a training history of at least 5 years in structured badminton practice. Prior to enrollment, each athlete underwent a comprehensive medical screening to verify freedom from musculoskeletal injuries or chronic health conditions that would contraindicate high-intensity exertion. Participant training/performance caliber. According to the Participant Classification Framework proposed by McKay et al. (2022), our participants can be classified as Tier 2 (Trained/Developmental), given that they were competitive collegiate badminton players with ≥5 years of structured training and regular participation in provincial-level competition. While all participants were familiar with intermittent high-intensity efforts, those who had engaged in specific structured HIIT or RST programs within the 3 months preceding the study were excluded to ensure a uniform baseline. Using a randomized design, participants were allocated into two equal cohorts: the Repeated Sprint Training group (RST, *n* = 14) and the High-Intensity Interval Training group (HIIT, *n* = 14) (see Fig. 1). Detailed demographic characteristics, including age, height, weight, and BMI, showed no significant baseline differences between groups and are presented in Table 1. The study protocol received formal approval from the Ethics and Research Committee of Shandong University (Protocol ID: IRB-SBMS-SDU(L)2025-01-18) and was conducted in strict adherence to the ethical standards of the Declaration of Helsinki. Written informed consent was obtained from all individuals prior to the commencement of data collection.

**Table 1:** The descriptive characteristics of the participants.

	Age (years)	Height (cm)	Weight (kg)	BMI
RST (*n* = 14)	19.79 ± 2.01	176.57 ± 4.78	70.03 ± 2.35	22.49 ± 1.10
HIIT (*n* = 14)	20.7 ± 1.4	177.64 ± 4.20	71.60 ± 3.85	22.69 ± 0.91

### Procedures

A randomized controlled trial design was employed, with group allocation performed *via* computerized randomization to prevent selection bias. Data collection occurred over four distinct sessions to assess cardiorespiratory fitness and badminton-specific motor skills. The testing sequence was: (1) graded exercise test for maximum oxygen uptake ( ${\dot {\rm V}}$O_2max_); (2) repeated sprint ability assessment; (3) vertical jump performance; and (4) change of direction (COD) speed. To ensure data validity, participants were advised to refrain from alcohol, caffeine, and strenuous exercise for 24 h preceding each visit. Additionally, dietary consistency was controlled by requiring participants to maintain a food diary for 3 days prior to baseline testing and to replicate this intake before post-intervention assessments. The 8-week training intervention commenced 48 h after baseline testing, with post-tests conducted 48 h after the final session under identical conditions. Adverse events and training-related injuries were monitored throughout the intervention. During each supervised session, participants were asked to report any pain, discomfort, or injury symptoms. In addition, weekly check-ins were conducted to document any adverse events, missed sessions due to illness/injury, training modifications, or medical consultations. All reported events were recorded by the investigators. Due to logistical constraints, assessors were not blinded to group allocation during post-testing. To minimize potential measurement bias, all assessments were performed using standardized procedures, identical verbal instructions, and consistent testing conditions at pre- and post-testing.

### Aerobic ability test

Aerobic fitness was evaluated using a graded exercise test (Zhao & Lu, 2024). Prior to testing, athletes completed a standardized 10-min warm-up consisting of 5 min of light jogging followed by 5 min of dynamic stretching. The graded test was performed on a treadmill (Technogym, Cesena, Italy), commencing at a speed of 8 km·h^−1^. The velocity increased by 1 km·h^−1^ every 3 min. Between stages, 30-s relief intervals were provided to facilitate earlobe blood sampling for lactate measurement. Lactate sampling was collected for physiological monitoring during the graded test (to document the metabolic response across stages) and was not intended for lactate-threshold determination in the current study. The device measured ${\dot {\rm V}}$O_2max_, as well as the first and second ventilatory thresholds (VT_1_ and VT_2_), following standard criteria (Alejo et al., 2022). The minimal velocity that ${\dot {\rm V}}$O_2max_ elicited was established as v ${\dot {\rm V}}$O_2max_ if it could be maintained for a minimum of 1 min. Two independent researchers localized the first and second ventilatory thresholds (VT_1_ and VT_2_). The VT_2_ identification criterion was a continuous rise in the ${\dot {\rm V}}$_E_ equivalent for O_2_ ( ${\dot {\rm V}}$_E_ ${\dot {\rm V}}$O_2_^−1^) and the ${\dot {\rm V}}$_E_ equivalent for CO_2_ ( ${\dot {\rm V}}$_E_ ${\dot {\rm V}}$CO_2_^−1^) ratio curves in relation to the decrease in end-tidal O_2_ tension (P_ET_O_2_). The first ventilatory threshold (VT_1_) was also established as the point where an increase in ${\dot {\rm V}}$_E_ ${\dot {\rm V}}$O_2_^−1^ and P_ET_O_2_ occurred without a simultaneous rise in the ${\dot {\rm V}}$_E_ ${\dot {\rm V}}$CO_2_^−1^ (Alejo et al., 2022).

### Anaerobic power test

#### Wingate anaerobic test

The Wingate Anaerobic Test (WAnT) was employed to assess anaerobic power and capacity, a method recently applied to rugby league athletes (Pienaar & Coetzee, 2013). The test was conducted as per the method described by Bar-Or (1987). Key metrics derived included Peak Power (PP), Mean Power (MP), and Fatigue Index (FI). The 30-s maximal effort was conducted on a Monark cycle ergometer (Ergomedic 828E, Vansbro, Sweden). Prior to the main test, participants performed a specific warm-up: 5 min of low-intensity pedaling at a self-selected load and cadence, followed by 5 min at 60 rpm against a 2 kp load. During the second 5-min block, participants executed a maximal sprint for the final 5 s of each minute. Following a 2-min recovery interval, the WAnT commenced. Subjects pedaled at maximal velocity for 30 s against a constant resistance calculated as 7.5% of their body mass (Bar-Or, 1987). Instructions emphasized reaching peak rpm as quickly as possible and maintaining the highest possible cadence until completion. Four researchers provided verbal encouragement throughout the duration of the test.

### Repeated sprint test

The RST protocol comprised six maximal 30-m sprints interspersed with 30-s rest intervals. Sprint and recovery times were captured with 0.001-s accuracy using a photoelectric timing system (Alge-Timing Electronic, Vienna, Austria) connected to a digital chronometer. During recovery, participants decelerated and walked to the subsequent starting point. To optimize efficiency, dual timing gates functioned in opposite directions, allowing participants to begin the next sprint from the previous finish line, thus avoiding the need to return to the initial start. A standing start was utilized, with the lead foot positioned 30 cm behind the timing beam. Timing was triggered when the participant broke the light beam. Strong verbal encouragement was provided by researchers stationed at both ends of the track. Outcome measures included Ideal Sprint Time (IS), Total Sprint Time (TS), and Performance Decrement (PD). IS was calculated as the fastest 30-m sprint time multiplied by six, whereas TS was calculated as the sum of all six sprint times. PD, used as an indicator of fatigue, was calculated as ([TS/IS] × 100) − 100 (Spencer et al., 2006).

### Modified agility T-test

**Figure 2:**
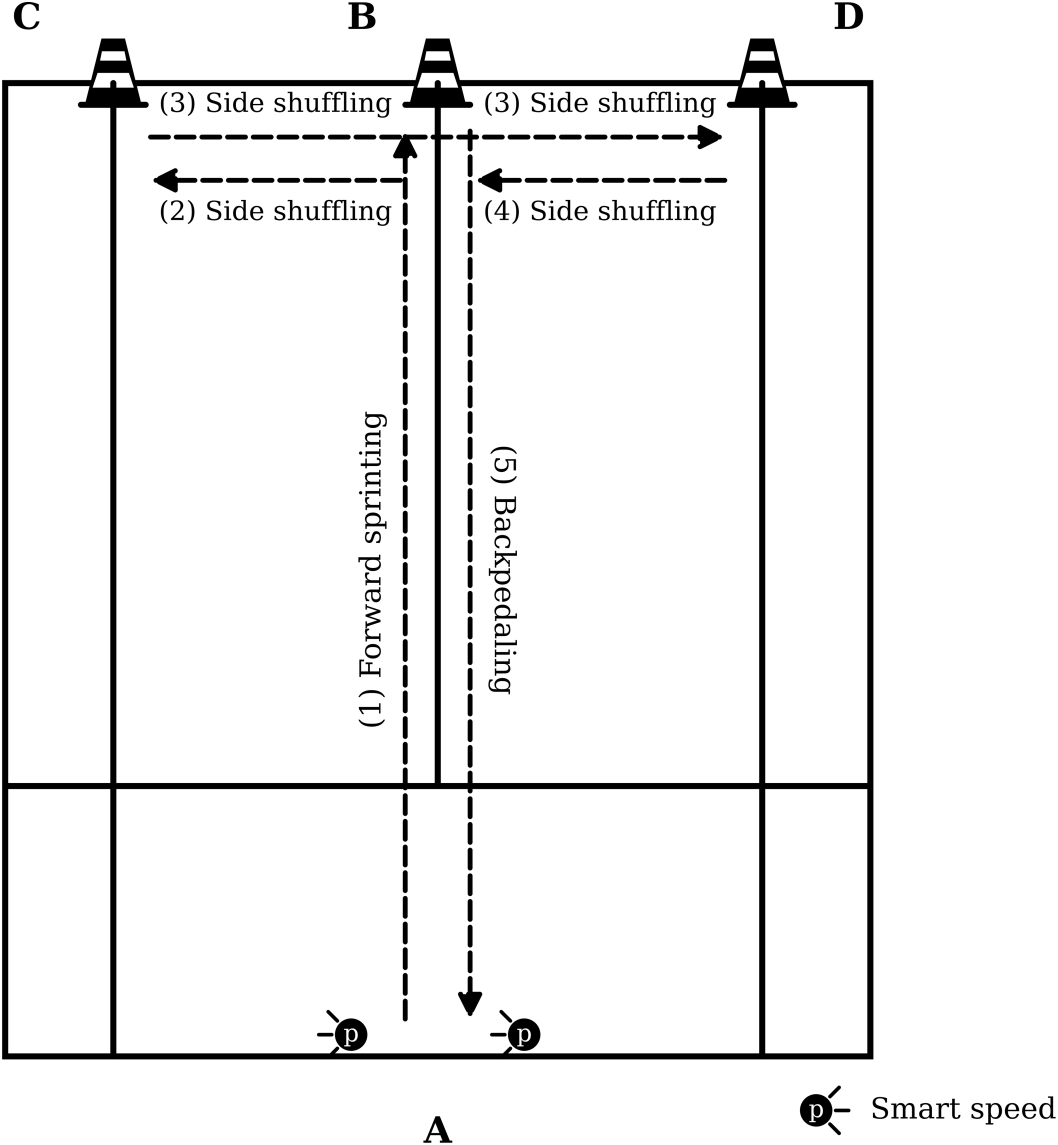
Modified agility T-Test.

The T-test was utilized to evaluate agility, a common metric in sports performance (Reiman & Manske, 2009). Distances were modified to reflect badminton court dimensions (Fig. 2). Cone A was positioned at the start, with Cone B placed 6 m forward. Cones C and D were arranged 3 m to the left and right of Cone B, respectively. A Smart Speed device (Fusion Sport, Coopers Plains, Australia) was stationed at Cone A. Upon the signal “Ready, go,” participants sprinted forward from A to B, touching Cone B with their left hand. They then shuffled laterally to Cone C (touching with the left hand), shuffled to the opposite side to Cone D (touching with the right hand), shuffled back to Cone B, and finally backpedaled to the start at Cone A. Three trials were performed, with the fastest time recorded as the final score. Four cones (height: 38 cm) were placed at the designated points to define the modified agility T-test course. The test has demonstrated high test–retest reliability (ICC: 0.92–0.95) in previous research and is therefore considered an appropriate field-based measure of change-of-direction performance (Sassi et al., 2009).

### Lower limb power ability

A wall-mounted Vertec device (Power System, Minnesota, TX, USA) was used to measure countermovement vertical jump (CMJ) and spike jump (SPJ) height. For the CMJ, participants performed a maximal vertical jump from a standing position. The protocol required knee flexion to a 90° angle (marked by an elastic band) while strictly prohibiting arm usage to isolate leg power (Rong & Xiu, 2024). CMJ height was assessed using a Vertec device. Participants performed a countermovement jump without an arm swing (hands maintained on the hips during the countermovement and take-off), and then reached upward with the dominant hand during flight to displace the highest possible vane. Standing reach height was measured prior to jumping, and jump height was calculated as the difference between the highest displaced vane and standing reach height. The test sequence involved standing, flexing the knees, and jumping maximally. The reliability coefficient (ICC) for CMJ was 0.95. The SPJ assessment began by measuring standing reach height. Participants then positioned themselves 3–4 m from the Vertec device. Upon command, they performed an approach run and executed a full spiking jump, striking the vanes with the palm of their hand. A standard four-step badminton approach was permitted. SPJ score was calculated as the difference between jump height and standing reach (Dell’Antonio et al., 2022). The ICC for SPJ was 0.93. Three maximal attempts were allowed for each test, separated by 30-s rest periods, with the best score selected for analysis.

### Training program

Training sessions started with ~15 min of skill-based badminton drills warm-up, jogging, full-body stretching, and submaximal jumps. Then, the players in the RST group performed all-out repeated-sprint bouts in addition to the regular badminton training, twice per week. The training protocol consisted of 30-m all-out running sprints. The training progression was based on the number of sets performed per week: two sets of 6 × 30-m in the first 2 weeks, three sets of 6 × 30-m from weeks three to five, and tapering back to two sets of 6 × 30-m from weeks six to eight. Training volume was reduced in the last phase to minimize the residual effect of neuromuscular fatigue.

The sprints were interspersed by 30 s of passive recovery, and each set was separated by 2 min of active recovery at low intensity (walking). The players in the HIIT group underwent their regular skill-based badminton training sessions (*i.e*., technical-tactical) combined with high-intensity interval training. The main part of the training session varied based on the training program followed by the participants (Table 2). Participants in the HIIT group completed running-based high-intensity interval training twice per week for 8 weeks in addition to regular badminton training. Each session consisted of 20-s work intervals at a target intensity of approximately ~90% HR_max, separated by 30 s recovery between repetitions. Participants performed 6 repetitions per series, with 2 min recovery between series. The program was periodized as follows: weeks 1–2: 2 series; weeks 3–5: 3 series; weeks 6–8: 2 series. Heart rate was continuously monitored, and running pace was adjusted to ensure participants reached the target intensity during each work interval. The complete HIIT prescription is provided in Table 2.

**Table 2:** The main part of the training session for repeated sprint training group and high-intensity interval training group.

Exercise	The first stage (wk 1–2)	The second stage (wk 3–5)	The third stage (wk 6–8)
Sprint training (twice a wk)	Intensity:	Intensity:	Intensity:
	30-m all-out sprints	30-m all-out sprints	30-m all-out sprints
	Volume:	Volume:	Volume:
	6 rep/series, 2 series	6 rep/series, 3 series	6 rep/series, 2 series
	Rest:	Rest:	Rest:
	30 s/rep, 2 min/series	30 s/rep, 2 min/series	30 s/rep, 2 min/series
High-intensity intermittent training (twice a wk)	Intensity:	Intensity:	Intensity:
	90% HRmax	90% HRmax	90% HRmax
	Volume:	Volume:	Volume:
	20 s * 6 rep/series, 2 series	20 s * 6 rep/series, 3 series	20 s * 6 rep/series, 2 series
	Rest:	Rest:	Rest:
	30 s/rep, 2 min/series	30 s/rep, 2 min/series	30 s/rep, 2 min/series

**Note:**

S, Second; wk, week. HIIT intensity control: running pace was adjusted using real-time HR feedback to achieve ~90% HRmax during work bouts.

The Polar Team2 System (Polar Electro Oy, Kempele, Finland) was used to monitor the heart rate of each player throughout each training session. Data were extracted using custom software (Polar Team2, Electro Oy, Kempele, Finland) to obtain maximum heart rate (HRmax), time spent in each HRmax zone, and Training Impulse (TRIMP). During HIIT sessions, real-time HR feedback was used to regulate running intensity to achieve the prescribed target (~90% HRmax) during the work bouts; if a player’s HR response was consistently below the target zone, running pace was increased in subsequent repetitions/sets, whereas pace was reduced if HR exceeded the intended zone or the player could not sustain the required intensity. TRIMP considers the training duration and intensity simultaneously, reflecting the comprehensive internal load. The method to determine the athlete’s TRIMP was based on the formula proposed by Edwards (1994), where the time in each HRmax zone is multiplied by the corresponding weighting factor for that zone and the results summated (Table 3). HRmax for each player was established using the peak value recorded during the maximal field tests.

**Table 3:** HRmax% zones and corresponding weighting factors.

Zone	Weighting factor	HRmax%	Zone
I	1	50–60%	I
II	2	60–70%	II
III	3	70–80%	III
IV	4	80–90%	IV
V	5	90–100%	V

### Statistical analysis

Data were analyzed using JASP software (version 0.18.3; JASP Team, Amsterdam, The Netherlands). Descriptive statistics (mean ± standard deviation) were calculated for all outcome measures. To determine the effects of the intervention, a two-way repeated measures analysis of variance (ANOVA) was employed, with group (RST *vs* HIIT) and time (pre- and post-intervention) as the independent factors. When a significant interaction was found, *post-hoc* comparisons with Bonferroni correction were performed to determine specific group differences. Effect sizes were reported using Cohen’s d with 95% confidence intervals (95% CIs) to assess the magnitude and uncertainty of the differences. Effect size thresholds were interpreted as trivial (<0.2), small (0.2–0.6), moderate (0.6–1.2), large (1.2–2.0), and very large (>2.0). An *a priori* power analysis was not performed before recruitment; thus, sample size was primarily based on feasibility and was comparable to other controlled training studies in similar athletic populations. To address statistical sensitivity, we conducted a *post-hoc* sensitivity analysis for the primary question of between-group differences in training-induced changes (post–pre). With *n* = 14 per group, a two-sided α = 0.05 provides 80% power to detect a large between-group effect on change scores (Cohen’s d ≈ 1.10). Therefore, the present study was adequately powered to identify large effects, whereas smaller between-group differences may not have been detected.

## Results

All participants completed the study, and their data were included in the final analysis. The Shapiro-Wilk test confirmed that all data were normally distributed (*p* > 0.286). Independent samples t-tests revealed no significant differences between the RST and HIIT groups at baseline for demographic characteristics (Table 1) or any of the physiological and performance variables (*p* ranges from 0.121 to 0.892). These results confirm the baseline equivalence of the two groups prior to the intervention. The primary endpoint was the change in modified agility T-test time from pre to post; all other outcomes were considered secondary. No adverse events or training-related injuries were reported in either group during the 8-week intervention.

**Figure 3:**
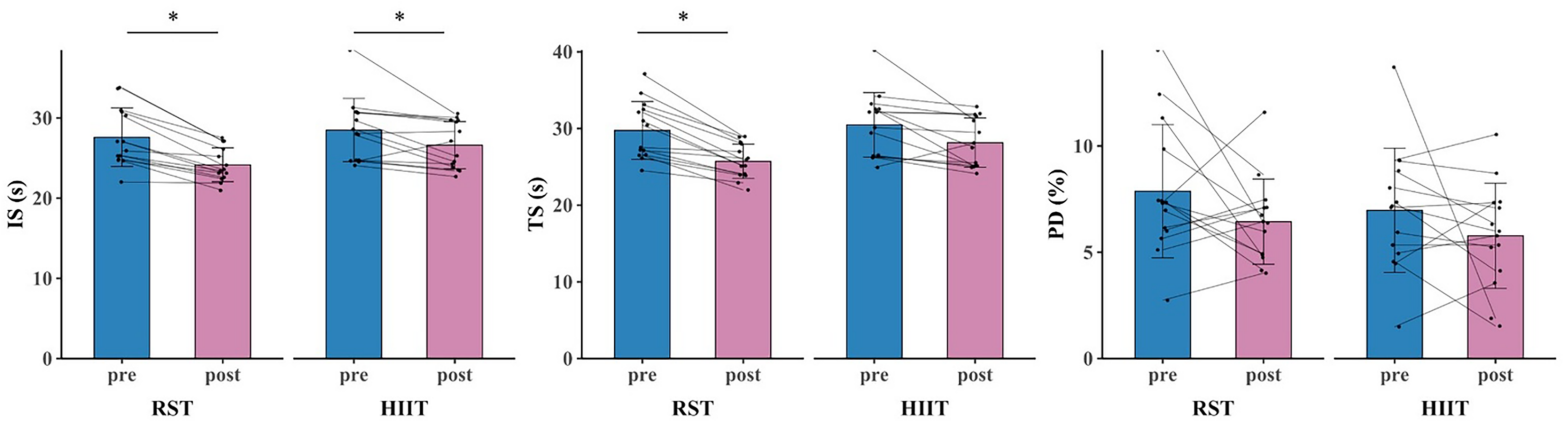
RSA test results for RST group and HIIT group before and after 8-week training. * denotes a significant difference between pre- and post-test (*p* < 0.05).

**Figure 4:**
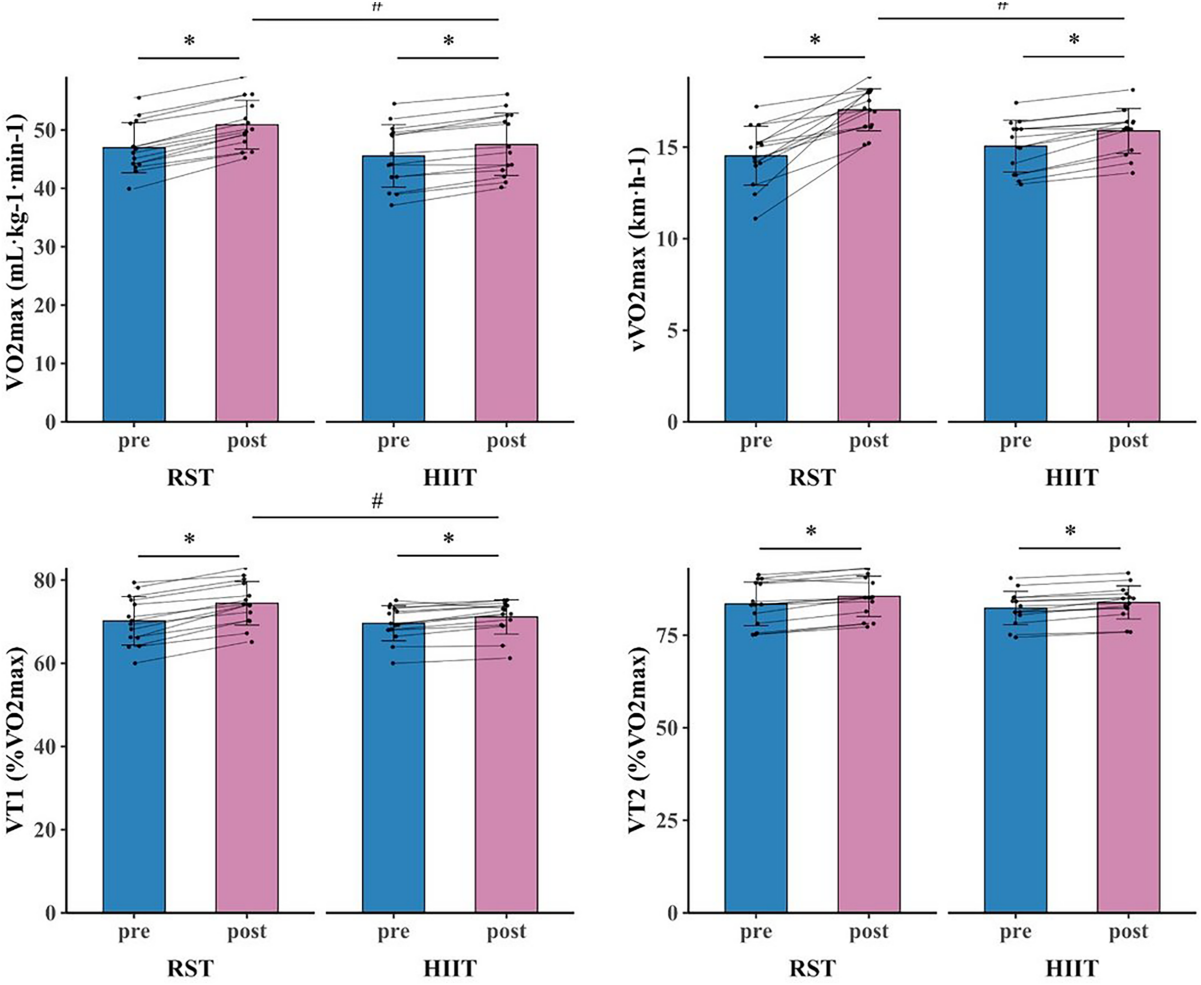
Aerobic ability test results for RST group and HIIT group before and after 8-week training. * denotes a significant difference between pre- and post-test (*p* < 0.05). # denotes a significant difference between RST group and HIIT group (*p* < 0.05).

**Figure 5:**
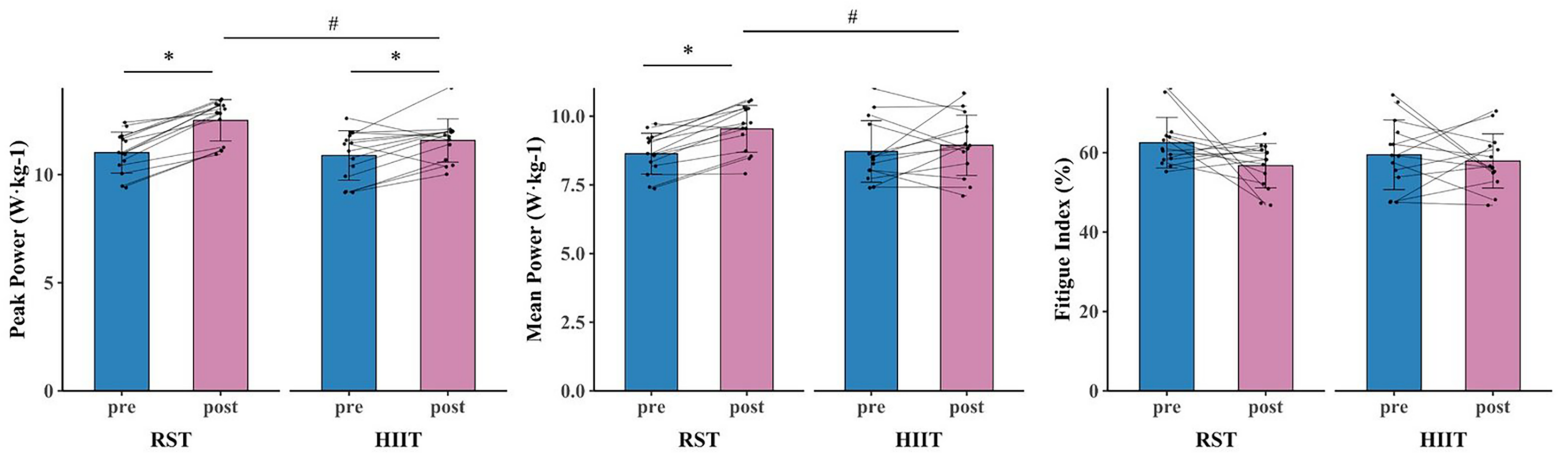
Anaerobic ability test results for RST group and HIIT group before and after 8-week training. * denotes a significant difference between pre-and post-test (*p* < 0.05). # denotes a significant difference between RST group and HIIT group (*p* < 0.05).

**Figure 6:**
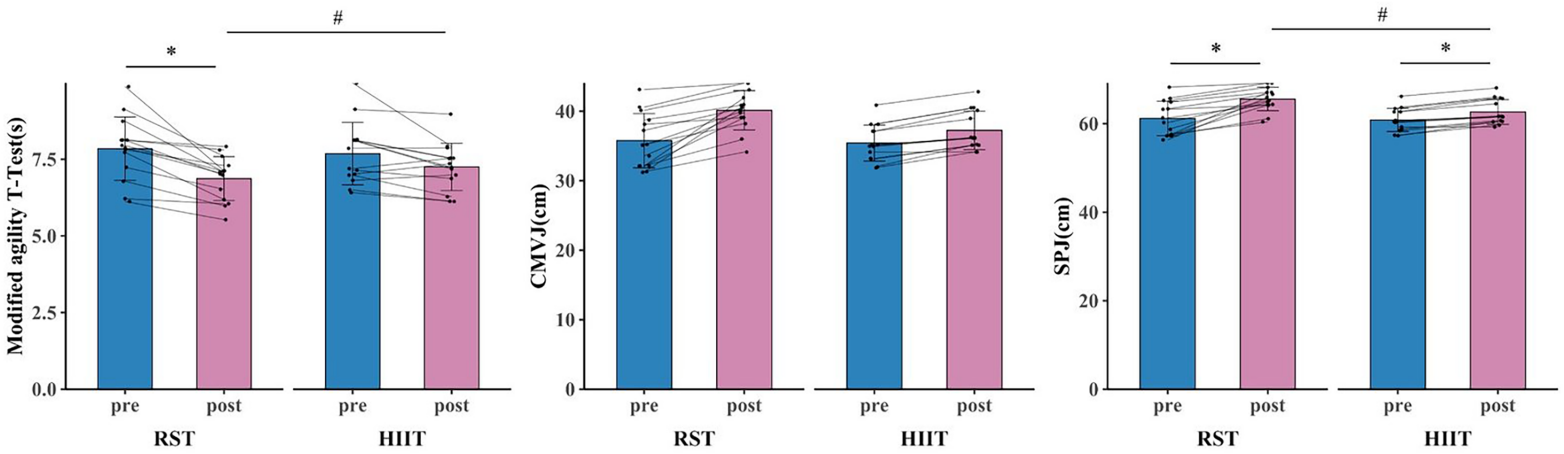
COD and lower limb power test results for RST group and HIIT group before and after 8-week training. * denotes a significant difference between pre- and post-test (*p* < 0.05). # denotes a significant difference between RST group and HIIT group (*p* < 0.05).

The primary two-way repeated-measures ANOVA models showed significant time and interactions between group and time on VO_2max_, vVO_2max_, VT_1_, PP, MP, modified agility T-Test, and SPJ. The *post hoc* analysis revealed that VO_2max_ (*p* < 0.001, d = 0.816 [0.467, 1.165]), vVO_2max_ (*p* < 0.001, d = 1.841 [0.911, 2.771]), VT1 (*p* < 0.001, d = 0.866 [0.452, 1.279]), PP (*p* < 0.001, d = 1.468 [0.649, 2.287]), MP (*p* = 0.002, d = 0.933 [0.196, 1.670]), modified agility T-Test (*p* < 0.001, d = 1.086 [0.387, 1.785]), and SPJ (*p* < 0.001, d = 1.438 [0.675, 2.201]) were significantly greater after RST intervention compared to all the other pre- and post- intervention. Additionally, within HIIT group, significant improvements in VO_2max_ (*p* < 0.001, d = 0.412 [0.202, 0.623]), vVO_2max_ (*p* = 0.049, d = 0.611 [0.031, 1.254]), VT1 (*p* = 0.005, d = 0.312 [0.049, 0.576]), PP (*p* = 0.020, d = 0.682 [0.036, 1.327]), and SPJ (*p* = 0.020, d = 0.597 [0.029, 1.165]) were observed after intervention as compared to baseline (Figs. 3, 4, 5, 6 and Table 4).

**Table 4:** The assessment results for RST group and HIIT group before and after 8-week training.

	RST group			HIIT group			Time effect	Time × Group interaction effect
	Pre (mean ± SD)	Post (mean ± SD)	Cohen’s d	Pre (mean ± SD)	Post (mean ± SD)	Cohen’s d		
IS (s)	27.60 ± 3.65	24.16 ± 2.11*	1.060	28.50 ± 3.94	26.62 ± 2.95*	0.581	<0.001	0.086
TS (s)	29.74 ± 3.76	25.72 ± 2.24*	1.172	30.48 ± 4.21	28.15 ± 3.21	0.677	<0.001	0.117
PD (%)	7.87 ± 3.14	6.44 ± 2.01	0.537	6.97 ± 2.93	5.77 ± 2.48	0.449	0.057	0.860
${\dot {\rm V}}$O_2max_ (mL·kg^−1^·min^−1^)	46.97 ± 4.30	50.90 ± 4.17*^#^	0.816	45.55 ± 5.34	47.53 ± 5.33*	0.412	<0.001	<0.001
v ${\dot {\rm V}}$O_2max_ (km·h^−1^)	14.53 ± 1.61	17.03 ± 1.15*^#^	1.841	15.05 ± 1.41	15.89 ± 1.23*	0.611	<0.001	<0.001
VT1 (%VO_2max_)	70.18 ± 5.83	74.43 ± 5.25*^#^	0.866	69.61 ± 4.22	71.14 ± 4.12*	0.312	<0.001	<0.001
VT2 (%VO_2max_)	83.46 ± 5.88	85.52 ± 5.45*	0.402	82.30 ± 4.51	83.85 ± 4.48*	0.304	<0.001	0.233
PP (W·kg^−1^)	11.02 ± 0.95	12.51 ± 0.96*^#^	1.468	10.89 ± 1.14	11.58 ± 1.00*	0.682	<0.001	0.014
MP (W·kg^−1^)	8.64 ± 0.74	9.54 ± 0.85*^#^	0.933	8.72 ± 1.12	8.94 ± 1.10	0.231	0.001	0.041
FI (%)	62.49 ± 6.38	56.73 ± 5.57	0.825	59.47 ± 8.77	57.89 ± 6.82	0.226	0.074	0.298
Modified agility T-Test (s)	7.85 ± 1.04	6.87 ± 0.72*^#^	1.086	7.68 ± 1.02	7.25 ± 0.77	0.480	<0.001	0.041
CMJ (cm)	35.77 ± 3.91	40.14 ± 2.82	1.424	35.43 ± 2.59	37.24 ± 2.78	0.591	<0.001	0.003
SPJ (cm)	61.20 ± 3.92	65.57 ± 2.64*^#^	1.438	60.84 ± 2.60	62.65 ± 2.80*	0.597	<0.001	0.003

**Notes:**

* Statistically significant difference between pre- and post-test, *p* < 0.05.

# Statistically significant difference between RST group and HIIT group, *p* < 0.05.

The primary two-way repeated-measures ANOVA models showed significant time effect on IS, TS, VT_2_, and CMJ. Within RST group, significant improvements in IS (*p* < 0.001, d = 1.060 [0.386, 1.734]), TS (*p* < 0.001, d = 1.172 [0.418, 1.925]), VT_2_ (*p* < 0.001, d = 0.402 [0.175, 0.628]), and CMJ (*p* < 0.001, d = 1.424 [0.668, 2.179]) were observed after intervention as compared to baseline. Additionally, within HIIT group, significant improvements in IS (*p* = 0.031, d = 0.581 [0.001, 1.160]), TS (*p* = 0.025, d = 0.677 [0.021, 1.334]), VT_2_ (*p* < 0.001, d = 0.304 [0.102, 0.505]), and CMJ (*p* = 0.020, d = 0.591 [0.029, 1.154]) were observed after intervention as compared to baseline (Figs. 3, 4, 5, 6 and Table 4).

## Discussion

The primary objective of this investigation was to contrast the physiological adaptations elicited by 8 weeks of RST *vs* HIIT in collegiate badminton players. This study extends previous findings by providing a comprehensive comparison that simultaneously evaluates metabolic thresholds, anaerobic power, and key neuromuscular qualities including agility and jump performance. Validating our hypothesis, the RST protocol yielded greater improvements across most of these physical capacities. These findings suggest that RST is a highly effective strategy for improving the underlying fitness determinants required for badminton, providing a robust physical foundation for match-play demands.

While both training modalities effectively improved repeated sprint performance (indicated by reduced Total Time), the RST group demonstrated larger effect sizes. This observation corroborates existing literature suggesting that sprint-specific interventions are particularly effective for enhancing RSA in intermittent sports (Zhao et al., 2024; Thurlow et al., 2023). The greater improvement observed in the RST cohort likely stem from physiological overload provided by the protocol rather than direct movement specificity. Although the 30-m linear sprint protocol exceeds the typical movement range of a badminton court (~13.4 m) and primarily assesses general linear sprint capacity rather than multidirectional agility, it appears to effectively enhance the underlying neuromuscular power required for match play. Additionally, time-motion analyses report average rally durations of approximately 6 s, a duration comparable to the 30-m sprints used in our protocol (Santiano et al., 2025; Green, West & Willems, 2023). Therefore, by targeting this specific metabolic timeframe, the improved general sprint capacity might effectively transfer to the anaerobic demands of court-based performance. Conversely, neither group exhibited statistically significant improvements in FI or PD. A plausible rationale is that since RST significantly improved RSAbest, the athletes were operating at a higher absolute power output during subsequent sprints. A higher power output inevitably induces greater metabolic perturbation and metabolite accumulation (*e.g*., H+ ions) per sprint. Therefore, the ability to maintain a similar FI despite the significantly increased anaerobic demand of faster sprints suggests that the training successfully enhanced neuromuscular power, even if the relative decline remains statistically unchanged.

Both RST and HIIT protocols resulted in significant within-group enhancements across aerobic parameters, including ${\dot {\rm V}}$O_2max_, v ${\dot {\rm V}}$O_2max_, VT_1_, and VT_2_. These findings align with prior research highlighting the robust cardiovascular adaptations stimulated by RST, particularly its efficacy in elevating maximal oxygen uptake and velocity at ${\dot {\rm V}}$O_2max_ (Ma et al., 2023; Sloth et al., 2013; Stankovic et al., 2023). Such physiological upgrades are critical for sustaining high-intensity efforts and accelerating recovery during the intermittent nature of badminton matches (Phomsoupha & Laffaye, 2015). Notably, while both groups showed significant improvements in VT_2_, no significant difference was found between the groups. This contrasts with previous evidence suggesting VT_2_ is highly sensitive to high-intensity endurance training (Liu et al., 2021). It is possible that the cumulative aerobic load and time-under-tension were similar between the protocols, providing a comparable stimulus for VT_2_ adaptation. This suggests that improvements in the second ventilatory threshold may represent a generalized response to high-intensity loading rather than a protocol-specific adaptation, particularly in this population.

Significant increments in PP and MP were recorded for both groups following the intervention. However, the RST group demonstrated greater magnitude of improvement in both metrics, supported by larger effect sizes and significant between-group differences. These results reinforce the consensus that maximal sprint-based training provides a more potent stimulus for the anaerobic energy system compared to submaximal intervals (Boullosa et al., 2022; Almquist et al., 2021). This suggests that while sprint training effectively elevates peak anaerobic output, its capacity to enhance tolerance to repeated maximal efforts may be limited within an 8-week timeframe. Adaptations related to buffering capacity typically necessitate longer training durations or higher cumulative loads. Furthermore, the participants’ status as trained athletes implies a higher baseline tolerance, potentially reducing the window for rapid improvement in fatigue resistance parameters.

A divergent response was observed in the modified agility T-Test, where the RST group achieved significant improvements, whereas the HIIT group did not. This finding is particularly intriguing given that the RST protocol consisted exclusively of linear sprinting. While agility development is often associated with multidirectional drills (Born et al., 2016; Zhou et al., 2024), our data indicate a transfer effect from maximal linear sprinting to COD tasks. This transfer is likely mediated by enhancements in eccentric strength and neuromuscular drive. The rapid deceleration phase inherent in maximal sprinting trains the eccentric force capabilities required for efficient direction changes (Young, Miller & Talpey, 2015). Additionally, the high-velocity contractions inherent to RST may have improved the rate of force development (RFD), thereby enhancing the re-acceleration phase during the agility test. These outcomes mirror findings by Kahraman & Kızılca (2025), who similarly reported agility gains following linear sprint training in youth athletes.

Consistent with the agility outcomes, the RST intervention produced significantly greater improvements in CMJ and SPJ height compared to HIIT. This disparity may be attributed to the biomechanical differences between running and the specific HIIT modality used; sprinting involves a high-velocity SSC akin to plyometric exercise. The substantial ground-reaction forces generated during maximal sprinting likely facilitated superior adaptations in leg stiffness and reactive power (Thurlow et al., 2023). This finding is particularly relevant for badminton, where vertical jump ability is a key physical determinant for the jump smash (Ma et al., 2025).

Certain limitations of this study warrant consideration. First, although internal load was monitored using heart rate and TRIMP, we did not conduct a systematic longitudinal comparison of internal training load between groups; therefore, we cannot confirm whether the overall physiological stress imposed by RST and HIIT was equivalent, and future work should incorporate detailed between-group internal-load quantification to better interpret adaptive differences. Second, while standardized laboratory measures were prioritized, no sport-specific endurance field tests were included; future studies should integrate ecologically valid assessments that more closely replicate match demands. Third, the sample comprised only male university-level players, which improves homogeneity but limits generalizability to female athletes, youth players, or less-trained populations. Fourth, there was methodological overlap between the RSA testing and the repeated-sprint training protocol, raising the possibility of training–testing overlap; thus, observed improvements may partly reflect task-specific adaptation or familiarization, and future research should include multidirectional repeated-sprint assessments to better evaluate sport-specific transfer. Fifth, assessors were not blinded to group allocation, which may have introduced measurement bias, particularly for agility and jump outcomes that are sensitive to tester expectations and encouragement. Finally, no *a priori* power analysis was performed; although the sample size (*n* = 28) is typical for training interventions, the study may have been underpowered to detect small-to-moderate between-group differences across the large set of outcomes assessed.

## Conclusion

In summary, this study found that integrating an 8-week, twice-weekly 30-m repeated sprint training program proved to be a highly effective strategy for concurrently improving aerobic capacity, anaerobic power, agility, and lower-limb explosive strength in male collegiate badminton players. Both training methods improved repeated sprint ability, but RST showed larger effect sizes across most indicators. These results suggest that this specific RST protocol is a more effective and time-efficient conditioning strategy for collegiate badminton athletes. Future studies should examine its long-term effects and applicability to other populations.

## Supplemental Information

10.7717/peerj.21111/supp-1 Supplemental Information 1 Raw data.

## Additional Information and Declarations

### Data Availability

The following information was supplied regarding data availability:

The raw data is available in the Supplemental File.
